# Effect of providing risk information on undergoing cervical cancer screening: a randomized controlled trial

**DOI:** 10.1186/s13690-014-0055-7

**Published:** 2015-02-23

**Authors:** Hiroyuki Fujiwara, Akihiro Shimoda, Yoshiki Ishikawa, Akiyo Taneichi, Mai Ohashi, Yoshifumi Takahashi, Takahiro Koyanagi, Hiroyuki Morisawa, Suzuyo Takahashi, Naoto Sato, Shizuo Machida, Yuji Takei, Yasushi Saga, Mitsuaki Suzuki

**Affiliations:** Department of Obstetrics and Gynecology, Jichi Medical University, 3311-1 Yakushiji, Shimotsuke, Tochigi Japan; Department of Public Health, Cancer Scan, Shibuya-ku Tokyo, Japan; Department of Health and Social Behavior, School of Public Health, The University of Tokyo, Bunkyo-ku Tokyo, Japan

**Keywords:** Cervical cancer screening, Informed choice, Risk information, Printed client reminder, Organized screening

## Abstract

**Background:**

In Japan, the cervical cancer screening rate is extremely low. Towards improving the cervical cancer screening rate, encouraging eligible people to make an informed choice, which is a decision-making process that relies on beliefs informed by adequate information about the possible benefits and risks of screening, has attracted increased attention in the public health domain. However, there is concern that providing information on possible risks of screening might prevent deter from participating.

**Methods:**

In total, 1,912 women aged 20–39 years who had not participated in screening in the fiscal year were selected from a Japanese urban community setting. Participants were randomly divided into 3 groups. Group A received a printed reminder with information about the possible benefits of screening, group B received a printed reminder with information about possible benefits and risks, and group C received a printed reminder with simple information only (control group).

**Results:**

Out of 1,912 participants, 169 (8.8%) participated in cervical cancer screening. In the intervention groups, 137 (10.9%) participated in cervical cancer screening, compared to only 32 (4.9%) of the control group (p < 0.001). In addition, logistic regression analysis revealed that there was no significant difference in screening rate between group A and group B (p = 0.372).

**Conclusions:**

Providing information on the possible risks of screening may not prevent people from taking part in cervical cancer screening among a Japanese non-adherent population.

## Background

Cervical cancer is the one of the most common cancers among women [1]. It is estimated that 275,100 women died of cervical cancer in 2008 [1]. Further, the incidence of cervical cancer in Japan was 9,794 in 2008 [2]. Early detection of cervical cancer and its treatment is highly important because the 10-year survival rate for cervical cancer varies dramatically based on the stage of detection: from 15% (distant spread of the disease) to 93% (the disease is localized) [3]. In fact, the United Kingdom has achieved a 40% decrease in cervical cancer mortality rate, with the screening rate rising to over 85% [4]. Given that the cervical cancer screening rate in Japan is extremely low (32.7%, attendance rate in 2013) [5], increasing the screening rate is one of the most important public health issues.

Towards improving the cervical cancer screening rate, encouraging eligible people to make an informed choice—a decision-making process that relies on their beliefs informed by adequate information about the possible benefits and risks of screening—has attracted increased attention in the public health domain [6-10]. In fact, the 2012 Japanese cancer control plan mentions for the first time the promotion of possible risks of screening to participants [11].

While much attention has been devoted to informed choice, there has been concern that providing information on possible risks of screening might prevent people from participation. In fact, screening invitations generally do not include information about the major harms of screening. Jørgensen and Gøtzsche published a survey on the quality of information used in mammography screening programs [12], finding that the major harms of screening were not mentioned in any of the 31 invitations. In addition, Gigerenzer and colleagues recently reported a European survey on the continuing dramatic overestimation of the possible benefits of mammography and prostate-specific antigen testing in the vast majority of women and men [13]. In the article, the authors found that frequent consulting of physicians and health pamphlets tended to increase rather than reduce overestimation. A systematic review reports that most of the recent randomized controlled studies aiming to enhance informed choice resulted in no change in screening rate compared with usual care [14]. As such, enhancing informed choice might not have an adverse effect on screening participation [14]. However, this observation is based on findings for breast, colorectal, and prostate cancer screening, and the evidence regarding cervical cancer screening remains limited. Accordingly, the purpose of this study is to evaluate the effect of providing information on potential benefits and risks of screening via a printed reminder on cervical cancer screening rate among a non-adherent population.

## Methods

### Setting

This study was conducted in an urban area of Japan. The 2013 census revealed that approximately 250,000 people live in the area. An organized cervical cancer screening program has been provided by the local municipality. The local municipality’s website and monthly community newsletter that is mailed to residents offer information about the screening program. No other invitations were available previously. In the area, the provided screening program was an annual to triennial Pap smear and human papillomavirus (HPV) DNA testing for women over 20 years old. The interval between screening varies by test results. Participants with positive Pap smear results are offered extensive screening. If the results of the Pap smear are negative, participants with positive results of the HPV DNA testing are offered to take screening again in the next year. If both of the results are negative, the participants are offered to take screening after 3 years. Eligible individuals can be screened at the local medical association’s network of 8 clinics. More than 11,000 individuals, or around 21.3% of the eligible people in this community, participate in this cervical cancer screening program each year.

### Procedure

This study used a prospective randomized controlled design in a Japanese community setting. We selected women aged 20–39 years who had not participated in the screening in 2013 as non-adherent participants for this study.

**Figure 1:**
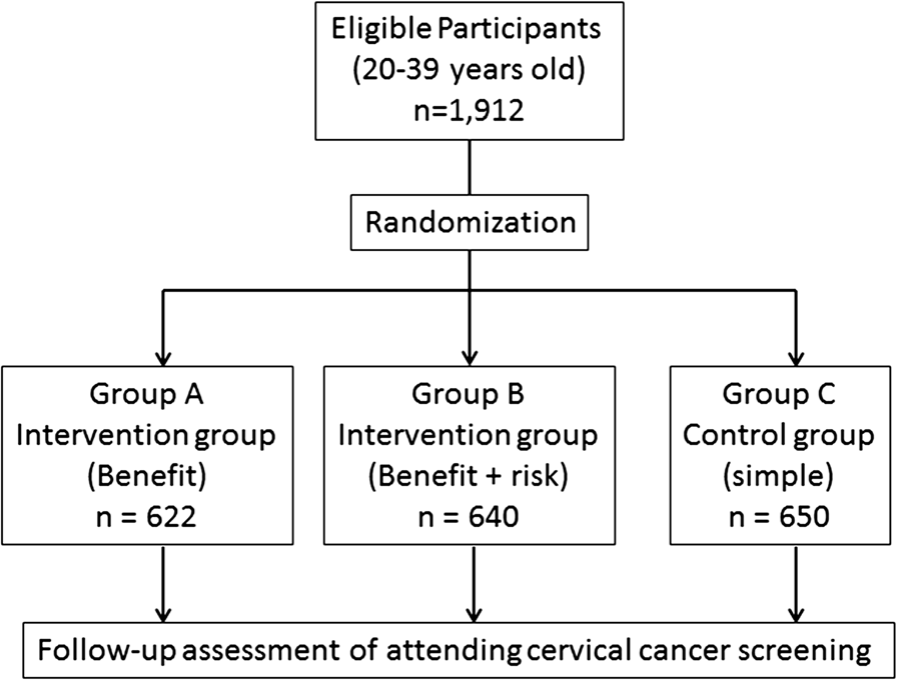
**Flow diagram of the trial process.**

Figure 1 shows the flow of participants in this study. In October 2013, the local health department’s database identified a total of 1,912 women aged 20–39 years who had not participated in the screening in 2013. Then, the 1,912 participants were randomly separated into 3 groups. Group A received a printed reminder with information on the possible benefits of screening (n = 622), group B received a printed reminder with information on the possible benefits and risks (n = 640), and group C received a printed reminder with simple information (control group, n = 650). Participants were randomly assigned to groups using a scheme that used blocks of random permutations of varying lengths and was stratified by age. As this study was conveyed as part of the local municipality-led health promotion program, the participant allocation ratio between the 3 groups was decided by the local municipality.

In November 2013, after the randomization, the local municipality sent the printed reminders via postal service to the 3 groups. Those who wanted to participate in cervical cancer screening were required to call either the local municipality or the designated local clinics to book the screening. The screening program was accessible until February 2014.

### Intervention

In this study, we conducted the intervention with printed reminders that included information about screening. Table 1 shows the messages received by each group. We used 3 messages as follows: for group A, we delivered a message including information about the possible benefits of screening. For group B, we delivered a message including information about the possible benefits and risks of screening. The message about possible benefits included information on susceptibility to cervical cancer, the 5-year survival rates for cervical cancer at each clinical stage, and the importance of early detection through screening. The message about possible benefits and risks included the above information, as well as information on the risk of false negatives, interval cancer, false positives, unnecessary treatment, and overdiagnosis. For group C, we delivered a message with simple information.

**Table 1:** **Messages received by each group**

**Group**	**Type of message sent**
Group A Intervention group (benefit)	● Susceptibility to cervical cancer.
	“The incidence rate of cervical cancer dramatically rises among individuals in their 20s and 30s”.
	● The respective 5-year survival rate of cervical cancer in each clinical stage.
	“The 5-year survival rate of cervical cancer is over 90% when the disease is localized”.
Group B Intervention group (benefit + risk)	Group B received the message including the above information, as well as information below.
	● Possibility of false negatives and interval cancer.
	“Accuracy of screening is not 100%. There is risk of interval cancer”.
	● Possibility of false positives and unnecessary treatment.
	“More than 90% of positive or suspicious patients will not be diagnosed as cervical cancer”.
	“You have risk for receiving unnecessary treatment”.
	● Possibility of overdiagosis.
	“It’s not sure whether detected cancer should be treated or not. You should talk with your doctor how to care the disease”.
Group C Control group (simple)	● Usual reminder.
	“You are due for your cervical cancer screening”.

### Data collection and survey measures

The main outcome was cervical cancer screening rate. Cervical cancer screening participation data were compiled as part of the usual record-keeping process of the designated local clinics. Each clinic sent a written notification to the local municipality when a cervical cancer screening had been taken. This information was then transferred to a medical history form and used to determine the participation in cervical cancer screening.

### Statistical analysis

Descriptive analysis was performed to summarize participants’ demographic characteristics. Logistic regression analysis with the control group serving as the reference group was conducted to determine whether cervical cancer screening participation differed between the intervention groups and the control group during a follow-up period of 4 months. We also conducted a logistic regression analysis to compare the effectiveness of the message between groups A (possible benefits of screening) and B (possible benefits and risks of screening). All analyses were based on intention-to-treat and were performed using IBM SPSS Statistics 19.0.

### Ethical issues

This study was approved by the Institutional Review Board (IRB) of Jichi Medical University and adhered to the Declaration of Helsinki. The IRB granted exemption of a written informed consent because of the minimal risk associated with the printed reminder and a guarantee by the local government of at least standard care for all eligible community members.

## Results

### Age and screening experience of participants

Table 2 reveals the age and screening experience among the groups. There were no significant differences between the 3 groups in the distribution of age and cervical cancer screening experience.

**Table 2:** **Age and screening experience among the groups**

	**Group A** **(n = ** **622):** **Intervention group** **(benefit)**	**Group B** **(n = ** **640):** **Intervention group** **(benefit + ** **risk)**	**Group C** **(n = ** **650):** **Control group** **(simple)**	**p-** **value**
Mean (SD) age (years)	29.6 (4.5)	31.4 (4.2)	31.0 (4.3)	0.413^a^
Age groups (years):				
20–29	270 (42.3)	295 (46.1)	275 (43.4)	0.372^b^
30–39	352 (57.7)	345 (53.9)	375 (56.6)	
Screening during 2 years before study	11 (1.7)	14 (2.2)	23 (3.4)	0.112^c^

**Values are presented as n** (%).

^a^p-value for comparison of age distribution between three groups by one-way ANOVA.

^b^p-value for comparison of age proportion between three groups by chi-squared test.

^c^p-value for comparison of cervical cancer screening experience between three groups by one-way ANOVA.

### Effect of intervention on cervical cancer screening rate

Out of 1,912 participants, 169 (8.8%) participated in cervical cancer screening. As shown in Table 3, 137 participants (10.9%) in the intervention groups participated in cervical cancer screening, whereas only 32 (4.9%) from the control group participated in screening (p < 0.001). In addition, as shown in Table 4, logistic regression analysis revealed that there was no significant difference in screening rate between groups A and B (p = 0.383).

**Table 3:** **Effect of intervention on cervical cancer screening rate**

	**Attendance n (%)**	**OR**	**95% CI**	**p value**
Group A	71 (11.4)	2.49	1.61–3.84	<0.001
Intervention group (benefit)				
Group B	66 (10.3)	2.22	1.43–3.84	<0.001
Intervention group (benefit + risk)				
Group C	32 (4.9)	1 (reference)	–	–
Control group (simpe)				

**Values are presented as n** (%).

*OR*: odds ratio, *CI*: confidential interval, p-value for comparison of cervical cancer screening rate between groups by logistic regression analysis.

**Table 4:** **Difference in effect of intervention by the type of message**

	**Attendance n (%)**	**OR**	**95%** **CI**	**p value**
Group A	71 (11.4)	1.17	0.82–1.66	<0.383
Intervention group (benefit)				
Group B	66 (10.3)	1 (reference)	–	–
Intervention group (benefit + risk)				

**Values are presented as n** (%).

*OR*: odds ratio, *CI*: confidential interval, p-value for comparison of cervical cancer screening rate between groups by logistic regression analysis.

## Discussion

Developing an effective method towards enhancing both informed choice and screening rate for cervical cancer is highly important in the public health domain. To our best knowledge, this is the first randomized controlled study to examine the effectiveness of an intervention providing risk information on screening attendance rate in cervical cancer screening.

The most important finding of this study is that providing information about potential risks of screening may not deter people from taking part in cervical cancer screening. Although previous studies have suggested that providing information about both risks and benefits of screening enhances informed choice, the effects on screening rate have been inconclusive [14]. Moreover, these studies have evaluated the effectiveness of interventions for breast, colorectal, and prostate cancer screening, and studies for cervical cancer screening have been limited. As one of the primary targets of cervical cancer screening is younger women with a low sense of urgency toward protecting their health, the effect of providing risk information to that population might be different when compared with the effectiveness among middle-aged and older adults, who have a greater sense of urgency with regard to preventing disease. As such, further studies are required to examine whether providing both benefit and risk information regarding cancer screening affects screening uptake.

The second important finding of this study is that interventions using printed reminders with additional information on possible benefits or risks of screening can be effective in improving cervical cancer screening rates among a non-adherent population (increase of 6.3 percentage points). Past studies have demonstrated that reminders are effective in enhancing cervical cancer screening rates (median increase of 10.2 percentage points: interquartile interval 6.3 to 17.9 percentage points; 14 study arms) [15]. However, non-adherent populations have been underrepresented in previous studies. While the benefit of early detection and treatment of cervical cancer has been established, non-adherence limits the value of these findings. As such, developing strategies to enhance the screening rate among non-adherent populations is of considerable public health importance. Further, few studies have examined the effect of printed reminders on cervical cancer screening among Asian populations [15,16]. As the effect of print reminders may vary across a range of settings and populations, further studies are required to examine strategies most effective among Asian populations towards increasing screening rate.

This study has several limitations. First, this study was conducted among women living in an urban area, so the results may not be applicable to groups of women in different settings. Second, the diversity of the collected data was limited, and thus we could not identify behavioral pathways. Additional data, including various psychological variables, will provide further information about reasons for responding or not responding to the printed reminders. Third, because we could not be certain of participants’ awareness of printed reminders among the intervention groups, we cannot be certain that the difference in cervical cancer screening rate between the groups resulted from the different information received by participants. Fourth, as this study did not measure the rate of informed choice, we cannot confirm that information on possible benefits and risks of screening enhanced the rate of informed choice.

## Conclusion

Providing information on the possible risks of screening may not prevent people from taking part in cervical cancer screening among a Japanese non-adherent population.
